# A Unique Case of Metformin-Associated Lactic Acidosis

**DOI:** 10.1155/2018/4696182

**Published:** 2018-11-15

**Authors:** Benjamin Gershkovich, Christopher McCudden, Kevin D. Burns

**Affiliations:** ^1^Department of Medicine, The Ottawa Hospital, University of Ottawa, Ottawa, ON, Canada; ^2^Department of Pathology and Laboratory Medicine, University of Ottawa, Ottawa, ON, Canada; ^3^Department of Medicine, Division of Nephrology, Kidney Research Centre, Ottawa Hospital Research Institute, University of Ottawa, Ottawa, ON, Canada

## Abstract

Metformin-associated lactic acidosis [MALA] is a potentially fatal condition characterized by an elevation in serum lactate in patients with metformin exposure. An 82-year-old man with no prior renal history was brought to hospital after being found by his family in a confused state. He had a history of type 2 diabetes mellitus, and his medications included regular metformin. On arrival to our hospital he was conscious but confused and noted recent decreased oral intake. Initial investigations revealed severe acidemia (pH <6.75, undetectable bicarbonate), with elevated serum lactate, urea, creatinine, and hyperkalemia. He was treated with intravenous dextrose, crystalloids, and bicarbonate and underwent urgent hemodialysis. The patient responded well to supportive therapies and achieved full renal recovery one week after admission. He was discharged feeling well, with a new antihyperglycemic medication regimen. This case highlights the potential for life-threatening acidemia in cases of MALA. The case is further unique in that the patient was conscious and responded to questions on arrival, despite the serious metabolic disturbance, and recovered completely. From a safety standpoint, health care providers should advise and educate their patients about discontinuing metformin and other potentially harmful medications in the context of acute illness with volume contraction.

## 1. Introduction

Metformin, a medication of the biguanide class, is a commonly used first line oral agent for the treatment of type II diabetes mellitus [DM2] [1]. Metformin improves glycemic control in DM2 via several complex mechanisms, including an increase in peripheral insulin sensitivity, improvement in peripheral glucose uptake, and a decrease in hepatic gluconeogenesis [2–4]. Since metformin is primarily excreted in the urine, dose adjustments are recommended for estimated glomerular filtration rate levels of 45 mL/minute/1.73 m^2^ or less [5]. The most common adverse effects of chronic metformin use are gastrointestinal complaints, including nausea, bloating, and diarrhea [6].

The rarest and most dangerous complication of metformin use is metformin-associated lactic acidosis [MALA]. The incidence of MALA in patients without renal disease is low, with a reported estimate of 5 cases per 100,000 patient-years [7]. The pathophysiology of MALA is thought to be multifactorial in nature. In rat models, metformin has been shown to promote lactate production in enterocytes [8]. Additionally, given that lactate is one of the substrates for hepatic gluconeogenesis, an accumulation of lactate may occur when this process is inhibited by metformin [9].

Various reports have documented high mortality rates for MALA. A case series of 49 patients with MALA reported an overall mortality rate of 45% [10]. The same study found that the degree of serum lactate elevation and serum metformin concentration do not appear to play prognostic roles. A separate retrospective study, involving 42 patients admitted to the intensive care unit, concluded that MALA related to intentional metformin overdose portends a much more favorable prognosis compared with MALA related to incidental metformin accumulation with concurrent medical illness [11]. Higher mortality was associated with increased age, lower arterial pH, elevated prothrombin time, and need for mechanical ventilation and vasoactive medications.

Here we describe a case of MALA in an elderly patient, who presented with confusion and unremarkable vital signs, despite demonstrating a profound depression in arterial pH and serum bicarbonate concentration. The patient fully recovered with fluid resuscitation, bicarbonate administration, and institution of hemodialysis therapy.

## 2. Case Presentation

An 82-year-old man who lived alone was brought to a regional hospital from his home after being found by his family in a confused state. His past medical history was significant for DM2, hypertension, dyslipidemia, benign prostatic hyperplasia, and chronic back pain. His medications consisted of metformin 1000 mg p.o. BID, sitagliptin 50 mg p.o. BID, ramipril 10 mg p.o. daily, tamsulosin 0.4 mg p.o. daily, hydrochlorothiazide 25 mg p.o. daily, and meloxicam 7.5 mg p.o. daily. He had no known prior history of cardiac or renal disease, and baseline serum creatinine [Cr] was 79 *μ*mol/L.

On initial assessment, the patient was in no acute distress, although he was disoriented and confused [Glasgow Coma Scale 14]. He complained of mild nausea with recent decreased oral intake, but there was no history of diarrhea. He had no history of infectious symptoms, toxic ingestions, recent medication changes, or witnessed seizure activity. On physical examination he had normal cardiorespiratory findings and no focal neurologic signs. His initial vital signs were blood pressure 150/83 mm Hg, heart rate 124/min, respiratory rate 33/min, oxygen saturation 100% on room air, and temperature 34.9° Celsius. His initial bloodwork results revealed a profound metabolic acidosis and acute kidney injury [Table 1].

A chest X-ray was unremarkable, and his electrocardiogram showed a wide QRS complex, prolonged PR interval, and peaked T waves.

He was initially treated with intravenous [i.v.] dextrose, a crystalloid bolus, and calcium gluconate, and his potassium was shifted intracellularly with inhaled salbutamol and i.v insulin. He also received one ampule of i.v. sodium bicarbonate. Given his profound metabolic disturbances, he was urgently transferred by ambulance to the local tertiary care centre for expedited management and consideration of dialysis. Upon arrival to the Emergency Department of the tertiary care hospital, he was oriented to person and place but not time, and vital signs were: blood pressure 110/80 mm Hg, heart rate 80/min, respiratory rate 20/min, and oxygen saturation 100% on room air. Within 30 minutes of arrival his mean arterial pressure [MAP] dropped to <65 mm Hg, and he required norepinephrine and vasopressin infusions as well as consultation with the intensive care unit [ICU]. At this time he also received a bolus of 1.5 litres of normal saline, and his axillary temperature was measured as 32.1° Celsius. An arterial blood gas [ABG] revealed profound metabolic acidemia (Table 2).

Blood cultures and serum toxicology screen for ethanol, methanol, isopropanol, acetone, ethylene glycol, acetaminophen, salicylates, and tricyclic antidepressants were negative. His remaining metabolic workup, including plasma thyroid stimulating hormone and plasma cortisol, was normal. Computerized tomography of the head, abdomen, and pelvis were negative for acute findings with no evidence of urinary obstruction or hydronephrosis. An assay for serum metformin level was unfortunately not available at our institution.

The patient's level of consciousness remained stable, and he consistently protected his airway. He required infusion of vasoactive agents for approximately 20 hours to maintain adequate blood pressure. He was heated externally using warming blankets, and his urine output during the first 24 hours of admission was 205 mL. In addition to treatment with i.v. crystalloid and bicarbonate, he underwent urgent hemodialysis treatment [dialysate HCO_3_^−^ concentration of 36 mEq/L] via central venous access, which was repeated the next day. After two days he was transferred out of the ICU and went on to achieve renal recovery [serum Cr 95 *μ*mol/L on day 8 of hospitalization] with restoration of urine output, acid-base and electrolyte balance. His functional status at the time of discharge was at his baseline. The metformin was discontinued from the day of admission, and he was prescribed a new regimen of oral antihyperglycemic medications for his diabetes.

## 3. Discussion

This patient presented with acute kidney injury associated with severe hyperkalemia, hypoglycemia, and a profound metabolic acidemia with elevated anion gap and serum lactate levels. The patient's high anion gap metabolic acidosis was likely due to lactic acidosis and acute kidney injury, and he demonstrated appropriate respiratory compensation. Despite having severely depressed pH and HCO_3_^−^ levels however, the patient maintained consciousness throughout and did not require ventilatory support. Although a serum metformin assay was not available at our institution, other causes of elevated serum lactate including sepsis and tissue ischemia were ruled out, and there was no evidence of hepatic disease or toxic ingestions. Although the patient's transient hypotension may have contributed to his lactic acidosis, his serum lactate was significantly elevated on initial presentation, prior to the requirement of vasoactive agents. Overall, his presentation was in keeping with MALA.

In this case, it is unclear what precipitated the patient's rapid decline and initial presentation to hospital, as history was limited. However, based on his medication profile and laboratory findings, it is possible that he developed acute kidney injury secondary to extracellular volume contraction in the setting of nonsteroidal anti-inflammatory [meloxicam] and ACE inhibitor [ramipril] use, with resultant hypoglycemia and severe lactic acidosis secondary to metformin accumulation. Thus, this case illustrates the potential danger of volume contraction in elderly patients with DM2 who are treated with renin-angiotensin blockade, nonsteroidal anti-inflammatories and metformin, which can set off a cascade of events leading to life-threatening MALA.

This case was unique for several other reasons. Firstly, the patient's initial clinical appearance and findings on exam were not suggestive of critical illness, and very much out of keeping with his abnormal biochemical profile. Despite having undetectably low serum pH and bicarbonate values, he was able to maintain an acceptable level of mentation, and consistently protected his airway without the need for intubation. In this regard, a correlation exists between low pH and altered mental status, with progression to coma frequently seen in patients with serum pH <6.9 [12].

Additionally, although mortality rate is high in cases of MALA [10, 11], this patient had an unusually favorable clinical outcome. His advanced age, requirement of vasopressor therapy, elevated prothrombin time, and extent of acute kidney injury placed him at increased risk of death, yet remarkably he experienced renal recovery and had returned to his baseline functional status by the time of hospital discharge.

Hypothermia in MALA has been described previously in several case reports [13–15]. While there does not appear to be a direct relationship between metformin and thermoregulation, it has been hypothesized that hypothermia in cases of severe MALA may be a consequence of systemic vasodilation, which is induced by acidemia [13]. Hypoglycemia is also known to cause hypothermia [16] as a result of decreased available substrate for cellular respiration and heat production. In our patient, the combination of acidemia and hypoglycemia can explain his significant hypothermia, given that central, infectious, traumatic, and other endocrine causes of hypothermia were ruled out.

The patient's coagulopathy on presentation cannot be explained by liver disease, clotting factor deficiencies, or recent use of anticoagulant medications. It is possible that he may have developed vitamin K deficiency given his history of decreased oral intake. However, his INR and PTT corrected within 12 hours of admission, which is sooner than expected for reversal of a vitamin K deficiency. In human and animal models, acidemia has been shown to inhibit clot formation, which may be due to increased fibrinogen consumption [17, 18]. Additionally, hypothermia inhibits thrombin generation* in vitro *[19, 20], which may result in the clinical finding of elevated PT and PTT values. Therefore, it is most likely that our patient developed a coagulopathy as a result of the combined effects of hypothermia and acidemia, which corrected shortly after warming and treatment with bicarbonate and hemodialysis.

Finally, an interesting aspect of this case was the result of the ABG that was performed after the patient arrived at the tertiary care centre. The arterial pH and bicarbonate were reported as “<6.75” and “No result”, respectively. These values reflect the fact that the patient's true serum pH and bicarbonate were below the detectable limit for our institution's blood gas analyzer. On a typical blood gas analyzer, pH is measured by detecting the potential difference across a hydrogen-selective membrane. The potential difference is converted to hydrogen ion concentration using the Nernst equation. Bicarbonate is then calculated from pH and pCO_2_ using the Henderson-Hasselbalch equation [21].

In contrast, on a typical automated chemistry analyzer, bicarbonate measurement is based on a series of coupled enzyme reactions to convert bicarbonate to other compounds, such as malate. In the process there is a decrease in levels of nicotinamide adenine dinucleotide [NADH], which is measured spectrophotometrically by absorbance at 405 nm. The decrease in NADH concentration is then used to calculate the bicarbonate concentration, as there is a direct proportional relationship between the two variables [22]. Based on the Henderson-Hasselbalch equation, a serum bicarbonate concentration of 0 mmol/L is theoretically not possible. In fact, a set of serum electrolytes done at the time of the above blood gas confirmed a serum bicarbonate of 4 mmol/L in our patient, highlighting the need for vigilance in interpreting the ABG in such settings.

This case demonstrates the importance of early recognition of MALA in patients taking metformin who present with an unexplained high anion gap metabolic acidosis with elevated lactate levels. Such patients may present with clinical findings that do not correlate with their metabolic derangements and are at risk of rapid deterioration. Despite his advanced age and severe metabolic derangements, our patient had a very favorable outcome, perhaps as a result of prompt medical intervention, coupled with his relatively few medical comorbidities. Additionally, this case highlights the need for healthcare providers to educate patients about the importance of temporarily discontinuing metformin and other potentially nephrotoxic medications in the settings of acute illness and extracellular volume contraction.

## Figures and Tables

**Table 1:** Initial laboratory results [normal values in parentheses].

Hb [g/L]	130 [140-180]
WBC [x10^9^/L]	**17.3 **[4.0 – 11.0]
Platelets [x10^9^/L]	286 [150 – 400]
pH [Venous]	**6.85 **[7.33 – 7.46]
pCO_2_ [Venous] [mmHg]	**19.3 **[40.0 – 50.0]
HCO_3_^−^ [Venous] [mmol/L]	**3.4 **[22.0 – 27.0]
Na^+^ [mmol/L]	145 [135 – 145]
K^+^ [mmol/L]	**8.3 **[3.5 – 5.1]
Cl^−^ [mmol/L]	101 [100 – 110]
Anion Gap [mmol/L]	**40.6** [5 – 12]
Albumin [g/L]	28 [34 – 46]
Glucose [mmol/L]	**1.4 **[4.0 – 11.0]
Creatinine [*µ*mol/L]	**967 **[58 – 110]
Urea [mmol/L]	**31.2 **[2.5 – 7.0]
PO_4_^−2^ [mmol/L]	**3.68 **[0.80 – 1.45]
Ca^2+^ [mmol/L]	2.43 [2.20 – 2.52]
Mg^2+^ [mmol/L]	0.84 [0.65 – 0.90]
Bilirubin [*µ*mol/L]	6 [3 – 17]
ALT [Alanine Aminotransferase] [U/L]	22 [20 - 70]
AST [Aspartate Aminotransferase] [U/L]	32 [15 – 45]
ALP [Alkaline Phosphatase] [U/L]	44 [40 – 115]
aPTT [Activated Partial Thromboplastin Time] [s]	**45** [22-30]
PT [Prothrombin Time] [s]	**21.3** [10.1 – 14.6]
INR [International Normalized Ratio]	**1.9** [0.9 – 1.2]
Lipase [U/L]	351 [45 – 300]
Lactate [mmol/L]	**14.9 **[0.5 – 2.2]
CK [Creatinine Kinase] [U/L]	65 [30 – 200]
Troponin I [ng/L]	32 [<30]

**Table 2:** Arterial blood gas, serum lactate level, and serum electrolyte panel.

pH [Arterial]	**<6.75 **[7.38 – 7.46]
pCO_2_ [Arterial] [mmHg]	**<12 **[32 – 45]
HCO_3_^−^ [Arterial] [mmol/L]	**“No result” **[22 - 27]
Lactate [mmol/L]	**16.0 **[0.5 – 2.5]
Na^+^ [mmol/L]	142 [136 – 145]
K^+^ [mmol/L]	**5.6 **[3.5 – 5.1]
Cl^−^ [mmol/L]	107 [98 – 107]
Anion Gap [mmol/L]	**31 **[5 – 12]
Glucose [mmol/L]	13.7 [4.0 – 11.0]
Creatinine [*µ*mol/L]	**794 **[49 – 93]
Urea [mmol/L]	**33.0 **[2.1 – 8.0]
